# Drug Survival, Effectiveness and Safety of Secukinumab in Axial Spondyloarthritis up to 4 Years: A Real-Life Single Center Experience

**DOI:** 10.3390/jpm14040417

**Published:** 2024-04-15

**Authors:** Alexandra-Diana Diaconu, Cristina Pomîrleanu, Mara Russu, Georgiana Strugariu, Eugen Ancuța, Irina Ciortescu, Cristina Bologa, Bianca Codrina Morărașu, Mihai Constantin, Alexandr Ceasovschih, Victorița Șorodoc, Laurențiu Șorodoc, Codrina Ancuța

**Affiliations:** 1 Department of Internal Medicine, Faculty of Medicine, Grigore T. Popa University of Medicine and Pharmacy, 16 Universitatii Street, 700115 Iasi, Romania; alexandra-diana_diaconu@email.umfiasi.ro (A.-D.D.); cristina.bologa@umfiasi.ro (C.B.); codrina.morarasu@umfiasi.ro (B.C.M.); mihai.s.constantin@umfiasi.ro (M.C.); alexandr.ceasovschih@umfiasi.ro (A.C.); victorita.sorodoc@umfiasi.ro (V.Ș.); laurentiu.sorodoc@umfiasi.ro (L.Ș.); 2 Internal Medicine 2nd Department, ‘‘Sfântul Spiridon’’ Emergency Clinical County Hospital, 700111 Iasi, Romania; 3 Department of Rheumatology, Faculty of Medicine, Grigore T. Popa University of Medicine and Pharmacy, 16 Universitatii Street, 700115 Iasi, Romania; daniela.pomirleanu@umfiasi.ro (C.P.); chirica.mara@d.umfiasi.ro (M.R.); codrina.ancuta@umfiasi.ro (C.A.); 4 Rheumatoloy 2nd Department, Clinical Rehabilitation Hospital, 14 Pantelimon Halipa Street, 700661 Iasi, Romania; 5 Research Department, Elena Doamna Clinical Hospital, 700398 Iasi, Romania; eugen01ro@yahoo.com; 6 Gastroenterology Department, Faculty of Medicine, Grigore T. Popa University of Medicine and Pharmacy, 16 Universitatii Street, 700115 Iasi, Romania; 7 Institute of Gastroenterology and Hepatology, “Sfântul Spiridon” Emergency Clinical County Hospital, 700111 Iasi, Romania

**Keywords:** axial spondyloarthritis, secukinumab, drug survival, retention rate, dropout rate, biologic-naïve, biologic-experienced patients

## Abstract

(1) Objective: The main aims of our study were to explore the drug survival and effectiveness of secukinumab in patients with axial spondyloarthritis (axSpA). (2) Methods: We underwent a retrospective analysis of consecutive axSpA treated with secukinumab as a first line of biologics or at switch in a biologic-experienced population. Efficacy data, indicating improvement in inflammation parameters (such as C-reactive protein and erythrocyte sedimentation rate) and disease activity scores (such as Ankylosing Spondylitis Disease Activity Score [ASDAS-CRP], Bath Ankylosing Spondylitis Disease Activity Index [BASDAI]), and patient-reported outcomes (pain), were assessed at 6, 12, 24, 36 and 48 months. The drug survival rate, dropout rate and discontinuation reasons (efficacy versus safety) of secukinumab were assessed in subgroup analysis (axSpA with and without exposure to biologics). (3) Results: In total, 46 patients were exposed to the IL-17A inhibitor secukinumab. The drug survival for axSpA patients 59.7% at 12 months and 31.3% at 24 months. There were no statistically significant differences in the median drug survival between biologic-naïve versus biologic-experienced subgroups. (4) Conclusions: Secukinumab has demonstrated effectiveness and safety in treating a cohort of axSpA patients in real-world settings, with a notable retention rate of the drug.

## 1. Introduction

Spondyloarthritis (SpA) represent a group of chronic inflammatory conditions with an immune basis that are extremely heterogeneous but have common elements: genetic (*HLA-B27* and *non-HLA* genes), clinical (musculoskeletal and extra-articular involvement), imaging (sacroiliitis, spondylitis, peripheral arthritis, enthesitis, dactylitis) and therapeutic (good response to nonsteroidal anti-inflammatory drugs, NSAIDs) [1]. SpA include axial spondyloarthritis (axSpA)—ankylosing spondylitis (AS), non-radiographic axial spondyloarthritis (nr-axSpA) and peripheral SpA—reactive arthritis, arthritis/spondylitis associated with psoriasis (PsA) and arthritis/spondylitis associated with inflammatory bowel diseases [2].

AxSpA is a chronic autoimmune inflammatory disease predominantly affecting the axial skeleton and typically presenting with chronic low back pain and significant stiffness, which improves with physical activity [3].

Despite advancements in understanding axSpA, identifying it remains challenging, often causing diagnostic delays for patients. Ideally, diagnosis should precede the onset of radiographic sacroiliitis, but currently, the most sensitive imaging method for diagnosis is magnetic resonance imaging (MRI), which is expensive and not readily accessible to all.

The etiopathogenic mechanisms of axSpA are complex and incompletely elucidated, which raises the interest of researchers and practitioners for better management of the disease [4]. The key role in the pathobiology of axSpA is primarily influenced by two distinct inflammatory pathways: the tumor necrosis factor alpha (TNF-α) axis and the IL-23/IL-17A interleukin axis, with major implications in the specific pathogenic events (inflammation and ossification) that take place locally (axial and peripheral-articular or enthesitic) but also systemically (ocular, intestinal, skin) [5]. Recent studies have demonstrated the involvement of the intracellular JAK (Janus kinase) signaling pathway in the immune mechanisms of SpA, which creates the premises for a new therapeutic approach through JAK inhibition [6]. 

The management of patients with axSpA comprises both non-pharmacological and pharmacological interventions. The inadequate treatment of axSpA may result in notable disability, such as complete fusion of the axial skeleton, and a decrease in quality of life (QOL) [7]. For a long time, if a patient did not respond to non-steroidal anti-inflammatory drugs (NSAIDs), the only alternative options were tumor necrosis factor (TNF) inhibitors (TNFi). However, with the current availability of TNFi, along with interleukin-17 (IL-17) inhibitors (IL-17i) and Janus kinase (JAK) inhibitors (JAKi), there are now more therapeutic options and renewed hope for individuals living with this disease. Despite the existence of highly effective modern medications, making therapeutic decisions remains a challenge in current practice, necessitating further research. Biological therapy has significantly transformed the prognosis of SpA, often achieving therapeutic goals such as remission (REM) and low disease activity (LDA) in numerous cases. According to the European (European Alliance of Associations for Rheumatology, EULAR) and national (Romanian Society of Rheumatology) recommendations, the therapeutic approach in axSpA is standardized. Patients with active disease who do not respond, have contraindications or show adverse effects to NSAIDs are candidates for biologics as the first therapeutic line. Irrespective of their mechanism of action, all biologic drugs have demonstrated comparable efficacy in terms of articular manifestations; indeed, their use is prioritized based on SpA extraarticular manifestations such as uveitis and inflammatory bowel disease where monoclonal anti-TNF inhibitors are clearly superior versus etanercept as well as skin psoriasis where anti-IL-17 agents are recommended. Effectiveness of the chosen medication is carried out at predefined intervals (6 months) based on the new EULAR recommendations as well as National Therapeutic Guidelines [8,9]. 

However, approximately 40% of patients with SpA are not adequately controlled by the first biologic agent, and this is due to a lack of efficacy, eitherprimary non-responder (meaning that the patient has never responded to the administered medication) or secondary non-responder (the patient has lost response to treatment), or due to toxicity (the presence of adverse effects). Therapeutic failure (from either a lack of efficacy or toxicity) prompts a switch to another biologic agent, either with the same mechanism of action (cycling) or with a different mechanism of action (swapping). In patients experiencing sustained remission, tapering (reducing the dose or spacing out the interval of administration) can be considered, although the exact premises of how to carry out tapering (when, how, for what duration) are not precisely determined for patients with SpA [10].

Disease activity is monitored by internationally validated tools, namely BASDAI (Bath Ankylosing Spondylitis Activity Index) and ASDAS (Ankylosing Spondylitis Disease Activity Score), with the C-reactive protein (CRP) or ESR (erythrocyte sedimentation rate) variant, which assesses the current impact of SpA. Also, disease activity can be monitored by measuring one of the most common inflammatory biomarkers, CRP, with values in assessing radiographic progression [11].

Secukinumab (SCK), a fully human immunoglobulin G1 kappa monoclonal antibody targeting interleukin-17A, is a promising biologic disease-modifying antirheumatic drug (bDMARD), frequently recommended for the management of active axial spondyloarthritis (axSpA). Secukinumab functions by targeting IL-17A and preventing its interaction with the IL-17 receptor, which is expressed in various cell types, including keratinocytes. Secukinumab received approval for treating AS after proving its effectiveness across five multicenter phase III trials, including four randomized double-blind trials along with their extensions (MEASURE 1 [12], MEASURE 2 [12], MEASURE 3 [13], MEASURE 4 [14] and MEASURE 2-J [15]). Nevertheless, there are reservations regarding its use in individuals with inflammatory bowel disease (IBD) and uveitis [15,16].

The main aims of our study were to explore the long-term effectiveness of secukinumab in patients with axSpA in routine settings, to evaluate drug survival, to identify differences in clinical and laboratory assessments as well as the discontinuation rate in biologic-naïve vs. biologic-experienced patients and to document any adverse events that may have occurred within the cohort.

## 2. Materials and Methods

We performed a clinical audit of patients diagnosed with axSpA (fulfilling either ASAS 2009 criteria for axSpA or 1987 modified New York criteria for ankylosing spondylitis) treated with biologic agents (TNF inhibitors, IL-17 inhibitors) with regular follow-up in an academic rheumatology department during January 2015 and December 2023.

A total of 46 out of 195 axSpA patients in our registry of biologic therapy received secukinumab either as first line of biologics or at switch with at least 6 months of treatment and were enrolled in the current study, focusing on secukinumab prescription pattern in local settings, efficacy and safety data. 

We conducted a retrospective review of data collected at each monitoring visit every 6 months according to local recommendations and guidelines for the use of biological therapy in patients with non-radiographic axial spondyloarthritis and ankylosing spondylitis. 

We referred to parameters classically used in routine clinical practice as follows: (i) standard demographic data (age, gender, body mass index, smoking status, comorbidities); (ii) disease-related parameters including subtype of disease (non-radiographic or radiographic axSpA), disease duration, peripheral (arthritis, enthesitis, dactylitis) and extraarticular manifestations (anterior uveitis, psoriasis, inflammatory bowel disease), disease activity scores—BASDAI (Bath Ankylosing Spondylitis Index), ASDAS-CRP (Ankylosing Spondylitis Disease Activity Score calculated using C-reactive protein) and functional score BASFI (Bath Ankylosing Spondylitis Functional Index); (iii) treatment-related data such as previous biologic exposure (type and number of TNF inhibitors, time on biologics), duration of secukinumab exposure (months), drug discontinuation and reasons for treatment discontinuation (efficacy, safety).

Data were assessed at baseline and every 6 months, while efficacy results, including achieving the target of remission or low disease activity, drug survival rates, retention rate and dropout rates, were evaluated on annual basis for up to 4 years. 

### Statistical Analysis

Data were computed using SPSS 29.0. For qualitative data, frequency distributions were generated for the entire dataset and comparatively for stratification variables using contingency tables. For numerical data, standard descriptive statistical parameters were calculated, including mean, standard deviation, standard error of the mean, and minimum, maximum and median values.

We used significance tests, with the level of significance *p*-value = 0.05, for the comparative analysis of the values of the monitored variables in two or more groups. *p*-values were based on two-tailed testing. Beforehand, we checked whether the values of the analyzed parameter followed the normal distribution law using a fitting test, specifically the Kolmogorov–Smirnov test. Subsequently, we employed the Wilcoxon and Friedman tests for paired samples. In case of comparative analysis of numeric parameter values between different samples, we utilized the Student’s *t*-test and Mann–Whitney test along with their generalized versions (ANOVA and Kruskal–Wallis). For testing differences between the values of a qualitative variable across different batches, we employed the chi-square test. Kaplan–Meier curves were used to plot drug survival, while time to loss of response was analyzed using COX proportional hazard ratios, adjusting for age, previous use of other biologics and gender.

## 3. Results

### 3.1. Demographics and Disease-Related Parameters in Studied AxSpA Patients

Demographic parameters, clinical characteristics of axSpA patients, inflammatory parameters, disease activity and functional scores are summarized in Table 1. 

Up to two-thirds of the patients were male (71.7%), with a mean age of 52.48 ± 27.97 years and a mean disease duration of 15.39 ± 16.20 months, without significant differences among genders. 

All patients were likely to have high disease activity at baseline, as demonstrated by the mean BASDAI of 4.37 ± 2.02 and the mean ASDAS-CRP score of 3.10 ± 0.82.

All patients in our study benefited from non-steroidal anti-inflammatory drugs (NSAIDs) as the first therapeutic line, according to the European Alliance of Associations for Rheumatology recommendations. NSAIDs were discontinued in patients with active disease without therapeutic response, with contraindications or who experienced adverse effects. A total of 6 patients out of 46 in our study also benefited from methotrexate treatment, without a correlation with the patients’ evolution during secukinumab treatment.

### 3.2. Secukinumab Prescription Pattern in AxSpa Patients

Only four axSpA patients (8.7%) received secukinumab as the first-line biologic agent, while the majority (n = 42; 91.3%) were already exposed to at least one biologic agent and experienced treatment failure with one (n = 11; 23.9%), two (n = 20; 43.5%), three (n = 4; 15.2%), four (n = 7; 6.5%) and, even, five (n = 1; 2.2%) TNF-α inhibitors (data shown in Table 1). Adalimumab and etanercept were most frequently administered as first and second TNF inhibitors (in up to 40% of cases), while golimumab was likely to be recommended as the third or the fourth biologic agent (in 50% of cases); there is a local trend to use certolizumab in patients with multiple failures to either monoclonal anti-TNF antibodies or the etanercept, mainly because certolizumab has been reimbursed only the last years for axSpA patients according to local regulations.

The mean treatment exposure to 150 mg of secukinumab monthly was 15.39 ± 16.20 months, ranging from 6 to 60 months; a total of 14 (30.4%) out of 46 axSpA in our cohort required an optimized doze at 300 mg monthly to achieve the optimal disease control, with the therapeutic decision being adjusted as early as the 4th month of treatment and as late as the 34th month.

### 3.3. Secukinumab Efficacy Data

Disease activity scores, patients reported outcomes and inflammatory tests were evaluated every 6 months; the data at 6, 12, 24, 36 and 48 months are summarized in Table 2.

#### 3.3.1. Disease Activity Scores: BASDAI and ASDAS-CRP

- BASDAI score

**Figure 1:**
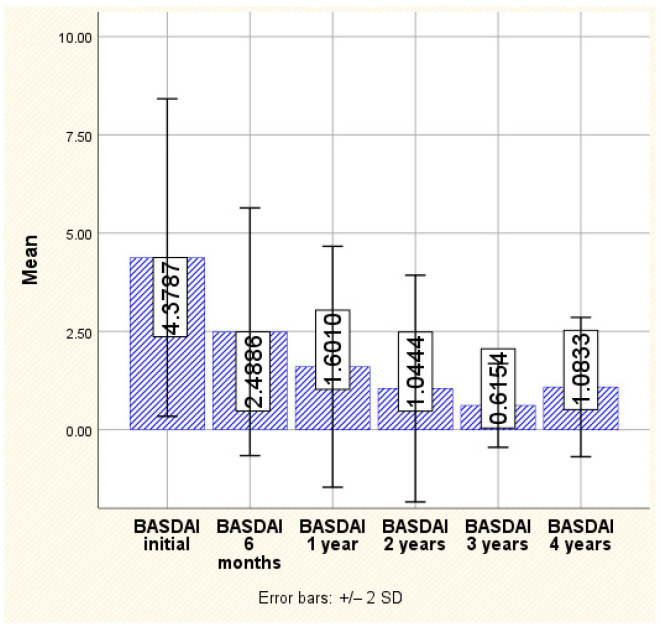
The evolution of BASDAI.

In the case of the BASDAI score, a continuous decrease is observed throughout the monitoring period, from the initial assessment where an average value of 4.37 ± 2.02 and a maximum of 9.20 were recorded to the last assessment, which was conducted 4 years after the initiation of treatment, when an average of 1.08 ± 0.88 and a maximum value of 2.80 were observed. This decrease is statistically significant, with notable reductions observed from one monitoring point to another (*p* < 0.001) (Figure 1).

- ASDAS-CRP score

**Figure 2:**
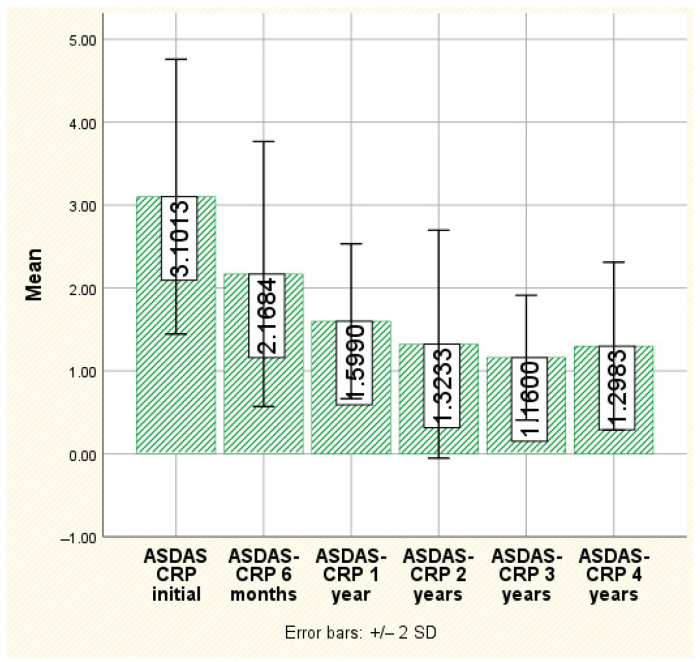
The evolution of ASDAS-CRP.

The ASDAS-CRP values show the same downward trend during the monitoring of their evolution over time, with statistically significant differences both globally and at each investigated time point (*p* < 0.001). Thus, initially, the average value of this score is 3.10 ± 0.82, with a maximum recorded at 5.75, and it decreases to an average of 1.29 ± 0.50 at 4 years after the initiation of treatment, with a maximum value of 2.09 (Figure 2). Both BASDAI and ASDAS-CRP values showed a statistically significant reduction between the start of secukinumab.

There were no statistically significant variances detected in BASDAI and ASDAS-CRP scores between the subgroup receiving the standard dosage of secukinumab and those administered with 300 mg of secukinumab. Likewise, no distinctions were noted in ESR and CRP levels within the same subgroup analysis. Similarly, there were no notable differences in BASDAI and ASDAS-CRP variations between biologic-naïve patients and those who had previously experienced TNF-α inhibition failure.

#### 3.3.2. Inflammatory Parameters (ESR and C-Reactive Protein)

- C-reactive protein

**Figure 3:**
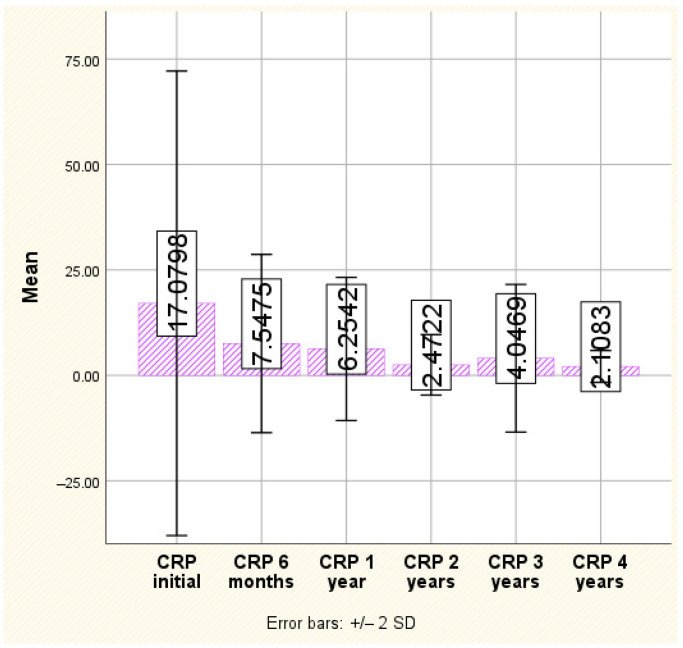
The evolution of CRP.

CRP serum concentration consistently decreased over time and showed a statistically significant improvement. Indeed, the average CRP at baseline was high (17.07 ± 27.53 mg/dL), with a maximum recorded at 132.00 mg/dL, with a marked decrease over time. At the 6-month evaluation, for example, it was less than half of the initial value, with an average of 7.54 ± 10.56 mg/dL, after which it continued to decrease. Although there was a slight revival at the 3-year evaluation (with an average of 4.04 ± 8.75, double that of the 2-year evaluation), at the 4-year evaluation, the decreasing trend resumed, with an average of only 2.10 ± 1.88 and a maximum of 1.60 (*p* < 0.001) (Figure 3).

- Erythrocyte sedimentation rate

**Figure 4:**
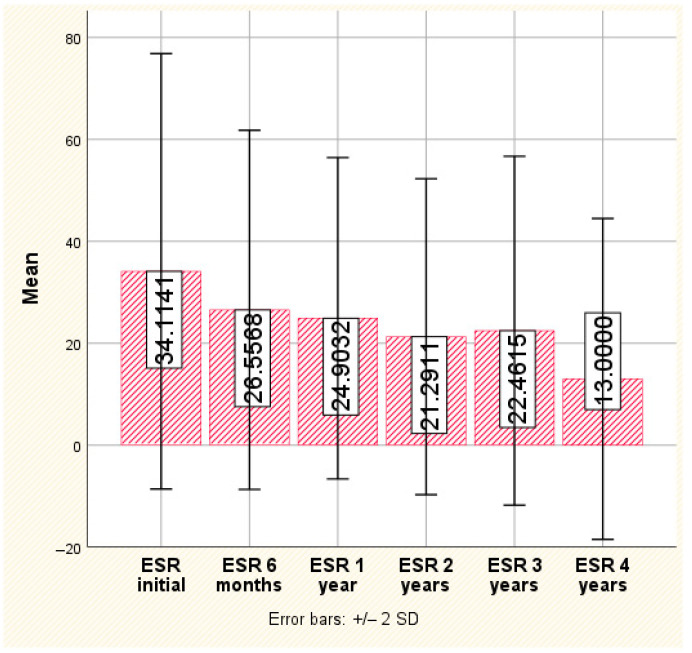
The evolution of ESR.

The same trend was reported for ESR; if initially, the ESR value was enough high to promote access to biologics, with an average of 34.11 ± 21.35 and a maximum of 96.0, we reported a consistent decrease at the 6-month, 1-year and 2-year evaluations, reaching an average of 21.29 ± 15.49 and a maximum of 52 (*p* < 0.001). The decreasing trend persisted at the 4-year evaluation (with the same slight resurgence observed at the 3-year evaluation), but this time, the recorded differences did not reach the threshold of statistical significance. Ultimately, the observed average value for ESR was 13.00 ± 15.73, with a maximum of 44, which is nearly three times lower than the value observed at initiation (Figure 4).

#### 3.3.3. Patient Reported Outcomes: Pain

Inflammatory back pain was initially rated as high by the majority of patients, with a mean VAS of 5.76 ± 1.56 and a maximum of 9.00; we demonstrated the same statistically significant decrease in pain score after 6 months, 1 year, 2 years and 3 years of secukinumab treatment, reaching a mean of 0.89 ± 0.64 after 4 years, value which is related to an optimal control of this patient-reported outcome measure (*p* < 0.001).

#### 3.3.4. Drug Survival Rate

The drug survival rate was 100% at 6 months, 59.7% in the first year, 31.3% in the second year, 20% in the third year and only of 8% at the end of the fourth year, respectively. There were no statistically significant differences in median drug survival between biologic-naïve versus biologic-experienced subgroups.

Up to 69.6 patients were treated with 150 mg of secukinumab monthly for up to 12 months, but only five patients maintained unchanged doses for more than 3 years (Table 3).

The average duration of treatment with 150 mg of secukinumab was 15.39 ± 16.20 months, ranging from 6 to 60 months. The dose was increased to 300 mg in 14 patients among the 46 treated (30.4%), with this therapeutic decision being adapted at the earliest in the 4th month of treatment and at the latest in the 34th month. In four cases (28.6%), 300 mg of secukinumab was administered over a period between 4 to 6 months. In another four cases (28.6%), a total of 300 mg of secukinumab was administered over 7 to 12 months. Another four patients received a high secukinumab dose for between 13 and 24 months, and two patients (14.3%) were given secukinumab for more than 25 months (25 and 34 months, respectively) (Table 4).

**Figure 5:**
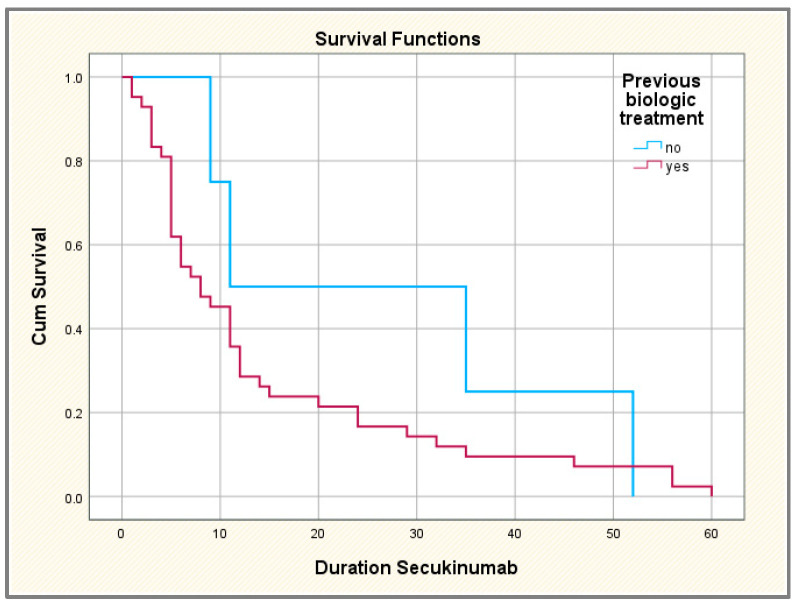
Comparative Kaplan–Meier survival curves in axSpA patients with/without previous exposure to biologics.

We compared the duration of treatment with secukinumab in patients with and without previous exposure to other biological agents but also based on the number of TNF inhibitors used (only one agent versus two or more agents); we used the Kaplan–Meier survival analysis and curves to plot median survival on IL-17 inhibitor Secukinumab (Figure 5). We have successfully demonstrated that the biologic-experienced population will discontinue secukinumab treatment faster compared to biologic-naïve patients; however, the difference between the two categories of patients was not statistically significant.

**Figure 6:**
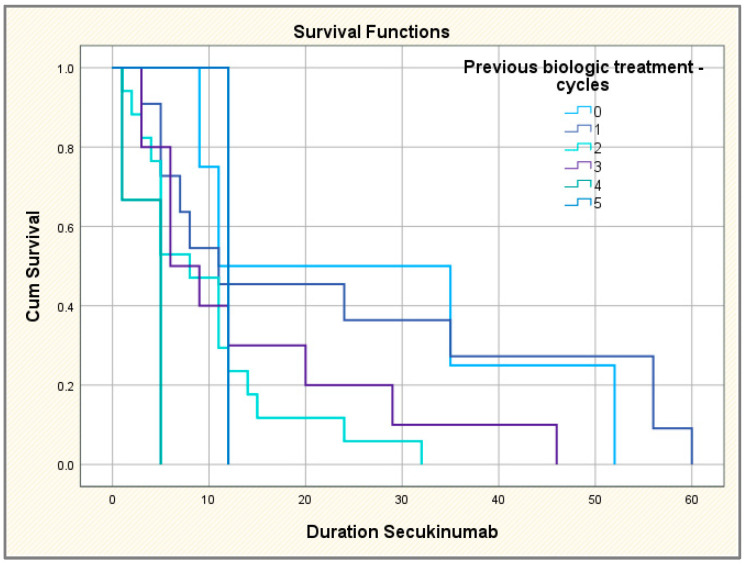
Comparative Kaplan–Meier survival curves according to the number of biologic drugs before exposure to secukinumab.

**Figure 7:**
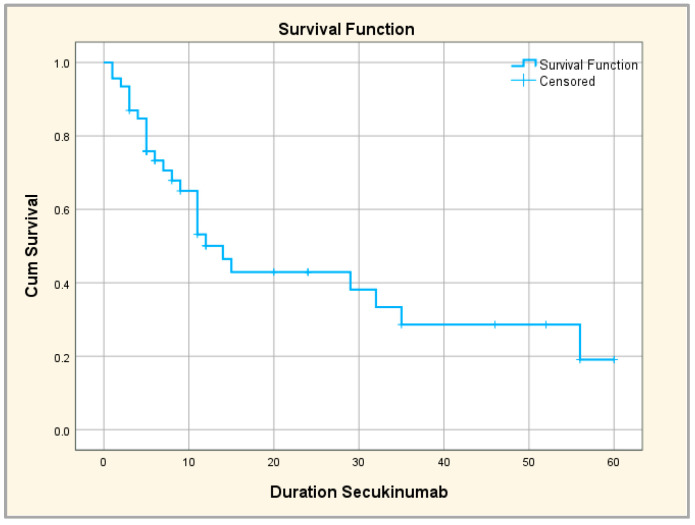
Global survival curve.

The comparative study of the survival curves according to the number of TNF inhibitors used highlights the same tendency: when continuing the treatment over a longer period of time, the more time passed, the fewer TNF inhibitors the patients had. Thus, the patients who continued the treatment for the longest time were those without previous biological treatment or with failure regarding only one TNF inhibitor; on the other hand, those who completed secukinumab treatment the fastest had at least four or five previous biological agents; the differences between categories of patients thus identified are statistically significant (Figure 6). We also performed the Kaplan–Meier survival curve to investigate the time of secukinumab administration until treatment discontinuation, overall and compared to patients with/without prior biologic treatment. The mean duration of secukinumab treatment (until discontinuation) was 25.7 months; a total of 25% of patients continued treatment for 56 months, 50% of patients continued for 14 months and 75% of patients continued for 6 months (Figure 7).

#### 3.3.5. Secukinumab Dropout Rate and Discontinuation Reasons

**Figure 8:**
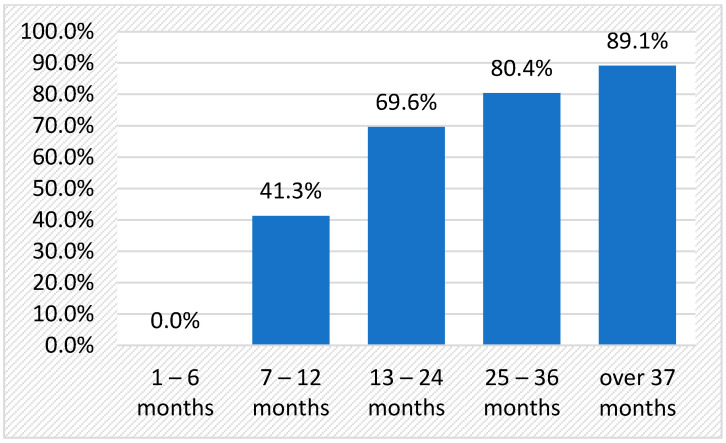
Secukinumab dropout rate in axSpA patients in long-term follow-up.

After four years of follow-up, only 44.5% (20 patients) remained on secukinumab, undergoing optimal disease control and no safety signals; the other 56.5% (26 patients) discontinued medication as early as after the first monitoring visit six months after initiation (Figure 8).

Clinical non-response (primary non-responders) or loss of therapeutic response (secondary non-responders) was the most common reason for treatment failure and subsequent discontinuation in 17.4% (8 cases) and 30.4% (14 cases), respectively. Adverse events, especially borderline paradoxical reactions, were the second main reason for drug cessation. In fact, two of the cases of Crohn’s disease reactivation during treatment with secukinumab also presented with flares of articular disease, resulting in mixed reasons for drug discontinuation; a third patient has developed de novo biopsy-proven Crohn’s disease after 12 months of secukinumab, leading to drug discontinuation. Furthermore, recurrent acute anterior alternate uveitis was reported in one male diagnosed with AS, finally resulting in drug interruption.

### 3.4. Secukinumab Safety Data

Apart from paradoxical adverse events (psoriasis reactivation), inflammatory bowel disease reactivation, de novo diagnosis of Crohn’s disease and recurrent anterior uveitis, no serious adverse events were reported during the four years of follow-up of secukinumab treatment.

No reactivation of latent infections (tuberculosis, B and C hepatitis) was reported, while less common side effects such as infections, injection site reactions and night sweats, although reported in some cases, were mild and did not induce any drug discontinuation.

## 4. Discussion

In recent years, the management of axSpA has undergone significant transformation with the introduction of biologic cytokine inhibitors such as TNF-α and IL-17 blockers. Our current study aims to offer insights into the long-term effectiveness and tolerability of secukinumab in patients with axSpA.

According to our findings, secukinumab resulted in a notable reduction in both ASDAS-CRP and BASDAI indices and VAS. Also, the laboratory markers CRP and ESR were significantly reduced compared to the initial values, similar to studies such as MEASURE and BIOBADASER [17,18]. These findings underscore the long-term efficacy of Secukinumab in addressing both clinical and laboratory manifestations of axSpA.

Our findings concerning the clinical response to secukinumab treatment aligned with our earlier short-term observations, as well as those reported in randomized controlled trials (RCTs) [19,20]. No differences in clinical response or retention rate were observed between naïve subjects and patients previously exposed to anti-TNF-α agents, as well as between patients receiving 150 mg/4 w of secukinumab vs. 300 mg/4 w of secukinumab. These results align with the outcomes observed in the MEASURE1 and MEASURE2 trials involving patients with axSpA, wherein secukinumab demonstrated efficacy in both biologic-naïve individuals and those who had prior exposure to anti-TNF-α agents [21,22].

We assessed statistically significant changes in ASDAS-CRP and BASDAI scores and in inflammatory markers (CRP and ESR) throughout the observation period. We also followed the assessment of the drug retention rate, the reduction of clinical and inflammatory markers between patients receiving 150 mg of secukinumab and those treated with 300 mg of secukinumab, the reduction of clinical and inflammatory markers between biologic-naïve patients and those previously treated with TNF-α inhibitors and the frequency of adverse events during the treatment.

Patients were treated with different treatments, as some patients were naïve to any biologic agent, while others initiated secukinumab after lack or loss of response to previous anti-TNF-α agents. Patients who had not been treated with TNFi before starting secukinumab were classified as TNFi-naïve, while those who had received one or more TNFi but did not respond effectively before initiating secukinumab were categorized as TNFi non-responders (TNF-IR).

Each patient in the cohort followed an induction scheme at the beginning of the treatment, which involved subcutaneously receiving 150 mg of secukinumab weekly for the initial five injections, followed by a maintenance dose of 150 mg every 4 weeks or 300 mg in primary non-responding patients.

We provide real-world data on the survival of IL-17Ai drugs in SpA and evaluate how diagnosis impacts drug survival. Alonso et al. presented data on secukinumab survival in axSpA and PsA from 154 patients in northern Spain, indicating an overall similar 1-year survival rate to our study (66% vs. 59.7%) and also a 2-year survival rate (43% vs. 31.3%), despite having a higher proportion of biologic-naïve participants [23]. The FORSYA multicenter study in France identified 904 patients with axSpA who were treated with secukinumab and found a 59% 1-year survival rate for axSpA, in congruence with our investigation [24]. Findings from the CANSPA network revealed a 12-month survival rate of 63% for 146 patients with axSpA [25].

Safety concerns were likely to correspond to those reported during randomized clinical trials, with no additional or significant safety signals. Overall, the long-term use of secukinumab was well tolerated in real-world settings.

According to published phase III studies, secukinumab does not raise the risk of uveitis [26]. In our study, only four patients experienced acute anterior uveitis under treatment with both secukinumab doses (150 mg monthly or 300 mg monthly), all featuring the first attack in the first years of their diagnosis of axSpA. Only one patient required a switch to another biologic agent: a 67-year-old male with a difficult-to-treat ankylosing spondylitis with multiple flares of uveitis long term before the use of secukinumab, even as a paradoxical event during adalimumab treatment; this patient suffered three more flares of bilateral uveitis during IL-17 inhibitor and required local intraocular glucocorticoids without complete recovery. The decision to stop secukinumab and to swap to certolizumab, as the only TNF inhibitor available, was taken, as the patient was also declared a secondary non-responder.

Several studies and case reports have indicated that secukinumab treatment can exacerbate inflammatory bowel disease [27,28]. In our study, two of the cases of Crohn’s disease reactivation were observed in the entire cohort, and a patient developed de novo biopsy-proven Crohn’s disease after 12 months of secukinumab.

Though the current analysis offers an accurate image of the real-life experience with secukinumab in a local cohort of axSpA treated with biologics, there may be some sources of limitation of our study, including (i) the retrospective review of medical records, even if data were collected according to a standardized protocol in compliance to biologic prescription registry; (ii) the sample size, especially referring to non-radiographic axSpA and biologic-naïve patients to secukinumab, which failed to allow a subgroup analysis and exact data able to reflect differences in drug survival and effectiveness in non-radiographic vs. radiographic axSpa and also in patients with previous exposure to biologic DMARDs vs. patients starting secukinumab as their first biological agent.

## 5. Conclusions

Secukinumab has demonstrated effectiveness and safety in managing a diverse cohort of axSpA patients in real-world settings, with a notable retention rate of the drug.

## Figures and Tables

**Table 1:** Demographics, disease-related and treatment-related characteristics at baseline.

Characteristics	AxSpA (n = 46)
Age (years) *	52.78 ± 14.23
Males, n (%)	71.7
Female–Male ratio	13:33
Smokers (current, ex), n (%)	4 (8.69)
BMI, median (IQR), kg/m^2^	27.89 ± 6.19
Disease duration (years) *	15.39 ± 16.20
Psoriasis, n (%)	1 (2.17%)
IBD, n (%)	2 (4.34%)
Uveitis, n (%)	4 (8.69%)
Previous anti-TNF failure, n (%)	42 (91.30%)
First-line biologic agent secukinumab, n (%)	4/46 (8.69%)
Failure with one TNF-α inhibitor, n (%)	11/46 (23.9%)
Failure with two TNF-α inhibitors, n (%)	20 (43.5%)
Failure with three TNF-α inhibitors, n (%)	4 (15.2%)
Failure with four TNF-α inhibitors, n (%)	7 (6.5%)
Failure with five TNF-α inhibitors, n (%)	1 (2.2%)
BASDAI *	4.37 ± 2.02
ASDAS-CRP *	3.10 ± 0.82
Pain (VAS 0–10) *	5.76 ± 1.56
CRP * (mg/dL)	17.07 ± 27.53
ESR * (mm/h)	34.11 ± 21.35

* Data expressed as mean ± SD; axSpA, axial spondyloarthritis; IQR, interquartile range; IBD, inflammatory bowel disease; SD, standard deviation.

**Table 2:** Efficacy data of secukinumab in axSpA up to 4 years of follow-up.

Activity Score	N		Mean	±SD	Wilcoxon Test
BASDAI	Initial		46	4.37	2.02
	6 months	44	2.48	1.57	*p* < 0.001 **
	1 year	31	1.60	1.53	*p* < 0.001 **
	2 years	18	1.04	1.44	*p* < 0.001 **
	3 years	13	0.61	0.53	*p* < 0.001 **
	4 years	6	1.083	0.88	*p* = 0.028 *
ASDAS-CRP	Initial		46	3.10	0.82
	6 months	44	2.16	0.79	*p* < 0.001 **
	1 year	31	1.59	0.46	*p* < 0.001 **
	2 years	18	1.32	0.68	*p* < 0.001 **
	3 years	13	1.16	0.37	*p* < 0.001 **
	4 years	6	1.29	0.50	*p* = 0.028 *
Laboratory Parameters					
CRP	Initial		46	17.07	27.53
	6 months	44	7.54	10.56	*p* < 0.001 **
	1 year	31	6.25	8.48	*p* < 0.001 **
	2 years	18	2.47	3.58	*p* < 0.001 **
	3 years	13	4.04	8.75	*p* < 0.001 **
	4 years	6	2.10	1.88	*p* = 0.028 *
ESR	Initial		46	17.07	21.35
	6 months	44	7.54	17.61	*p* < 0.001 **
	1 year	31	6.25	15.76	*p* < 0.001 **
	2 years	18	2.47	15.49	*p* < 0.001 **
	3 years	13	4.04	17.11	*p* < 0.001 **
	4 years	6	2.10	15.73	*p* = 0.028 *

Friedman test: Chi^2^ = 21.131; *p* > 0.001 *, *p* < 0.001 **; CRP, C reactive protein; ESR, Erythrocyte sedimentation rate; BASDAI, Bath Ankylosing Spondylitis Index; ASDAS-CRP, Ankylosing Spondylitis Disease Activity Score based on CRP.

**Table 3:** Time to discontinuation and dropout rate with secukinumab in axSpA.

	Duration of Treatment		Treatment Dropout Rate	
	N	%	N	%
1–6 months	19	41.3		0
7–12 months	13	28.3	19	41.3
13–24 months	5	10.9	32	69.6
25–36 months	4	8.7	37	80.4
>37 months	5	10.9	41	89.1
Total	46	100.0		

**Table 4:** Time to secukinumab dose optimization in partial responder axSpA patients.

		N	%		N	%
Dose increase 300 mg	no	32	69.6	1–6 months	4	28.6
	yes	14	30.4	7–12 months	4	28.6
				13–24 months	4	28.6
				25–36 months	2	14.3
	Total	46	100.0	Total	14	100.0

## Data Availability

Data are contained within the article.
